# Management and Treatment of Primary Membranous Nephropathy With a Positive PLA2R Marker

**DOI:** 10.7759/cureus.75057

**Published:** 2024-12-03

**Authors:** Rajesh Metuku, Austin B Wynn, Raul Santos

**Affiliations:** 1 Research Department, Alabama College of Osteopathic Medicine, Dothan, USA; 2 Nephrology, Archbold Hospital, Alabama College of Osteopathic Medicine, Thomasville, USA

**Keywords:** auto immune, end stage renal disease (esrd), idiopathic nephrotic syndrome, plar2, primary membranous nephropathy

## Abstract

Membranous nephropathy due to a positive PLA2R marker is an idiopathic cause of membrane nephropathy, characterized as an autoimmune attack on the kidney at the PLA2R receptor. Autoantibodies attack the PLA2R receptor, leading to nephrotic syndrome and eventually leading to end-stage renal failure, as in our case. We present a case that involves a patient who presented to the nephrology clinic with nephrotic range proteinuria and a history of HIV. The biopsy was prompted by nephrotic syndrome, where we saw clear evidence of sclerotic and fibrotic damage along with positive anti-PLA2R antibodies. The patient was put on tacrolimus and cyclophosphamide to halt the damage, but the patient eventually had to be put on dialysis later on. What makes this case unique is the patient is dealing with both the PLA2R antibodies and HIV, which increases the complexity of the treatment and our understanding of what played a bigger role in kidney failure. It is unique cases like these that prompt us to research further about these pathologies and develop new treatment options that result in a better prognosis.

## Introduction

The term “membranous nephropathy” gets its name from the pathological changes in the kidney where the glomerular basement is thickened due to the buildup of subepithelial immunoglobulin with minimum to no cellular proliferation. As such, it is a diagnosis to consider in nondiabetic patients with nephrotic syndrome. Membranous nephropathy can be further classified between primary or idiopathic causes or secondary causes due to various diseases such as SLE or medications [1]. The understanding of idiopathic membranous nephropathy was limited until the discovery of thrombospondin type-1 domain containing 7A or THSD7A and phospholipase A2 receptor (PLA2R), the protein involved in this patient. The PLA2R protein is normally found on the podocytes, and the disease starts when the immune system promotes an autoimmune reaction against an antigen in the podocyte, allowing subepithelial immunoglobulin build up and leading to kidney failure [1]. Nephrotic syndrome is one of the key findings of membranous nephropathy, but since it takes time for subepithelial immunoglobulins to build up and cause kidney damage, the symptoms of increased swelling and hyperlipidemia take time to develop, delaying a diagnosis.

The pathogenesis behind idiopathic membranous nephropathy is based on having immunoglobulins called G4, a type of IgG that is commonly found in the glomerulus. The anti-PLA2R antibodies colocalize the G4 immunoglobulins, which explains the nephrotic symptoms and the classification as an idiopathic cause. The PLA2R antibodies target the N-terminal cysteine with the dominant epitope to be within the three highest N-terminal concentration domains [2]. Further studies have shown that an estimated 80% of people are reactive to these epitopes from the 1st to the 7th C-type lectin-like domains, along with the three highest N-terminal concentration domains, which are referred to as epitope spreading [3].

The outcome of membranous nephropathy can depend on a variety of factors, such as whether it was caused by either a primary or secondary cause if the patient is treated properly, and if the patient even responds to the treatment. Some patients with normal kidney function and who lack any underlying disease can have spontaneous recovery, but patients with autoantibodies, like our patient, have a higher risk of progressing to end-stage renal disease (ESRD), which eventually did happen with our patient [4].

Treatment decisions are based on whether we are dealing with either primary or secondary in which primary membranous nephropathy can be treated with immunosuppressants such as tacrolimus and cyclophosphamide, as in our patient, and secondary causes are treated by targeting the initial insult. It is also important to know which marker a patient has since it can better tailor our treatment plans. Patients who have the PLA2R marker will benefit from immunosuppression medication such as cyclophosphamide due to expected renal failure [4]. By continuing to monitor the patient’s overall health and renal function, we will be able to better address any associated health problems with primary membranous nephropathy.

## Case presentation

A 35-year-old female with a past medical history of hyperlipidemia, HIV, and nephrotic range proteinuria was referred to the nephrology clinic due to worsening renal function, shown by the blood urea nitrogen and creatinine levels. An immune panel was significant for a CD4 count of 483 cells/uL (reference range of 490-1740 cells/uL), a positive ANA with a titer of 1:1280. The complete metabolic panel and urinalysis were ordered to better understand the state of the kidney, and the results were significant, with Table 1 for the CMP and Table 2 for the urinalysis, with reference ranges shown below.

**Table 1:** Complete metabolic panel. The complete metabolic table shows a large decrease in the glomerular filtration rate and an increase in creatinine from the reference values, indicating a substantial loss of kidney function.

Test	Patient value	Reference range
Sodium	140 mEq/L	136-145 mEq/L
Potassium	3.2 mEq/L (low)	3.5-5.1 mEq/L
Chloride	115 mEq/L (high)	98-107 mEq/L
Bicarbonate	16 mEq/L (low)	21-33 mEq/L
Blood urea nitrogen	19 mEq/L	6-20 mEq/L
Creatinine	1.8 mEq/L (high)	0.5-1.2 mEq/L
Glomerular filtration rate	33.90 mL/min (low)	>60 mL/min
Albumin	1.4 g/dL (low)	3.5-5.2 g/dL

The patient was referred initially due to an increase in lower limb edema and nephrotic range proteinuria of 500 mg/dL, shown in Table 2 below.

**Table 2:** Urinalysis The urinalysis is significant for a protein level of 500 mg/dL, which indicates proteinuria. Moderate urine hyaline casts are also indicative of a kidney pathology.

Test	Patient value	Reference range
Urine color	Yellow/hazy	-
Glucose	500	negative
Specific gravity	1.020	1-1.030
pH	6	5-8
Urine Protein	500 mg/dL	Negative
Urine white blood cells	9	0-2 /hpf
Urine red blood cells	3	0-2 /hpf
Urine hyaline cast	moderate	-

**Figure 1:**
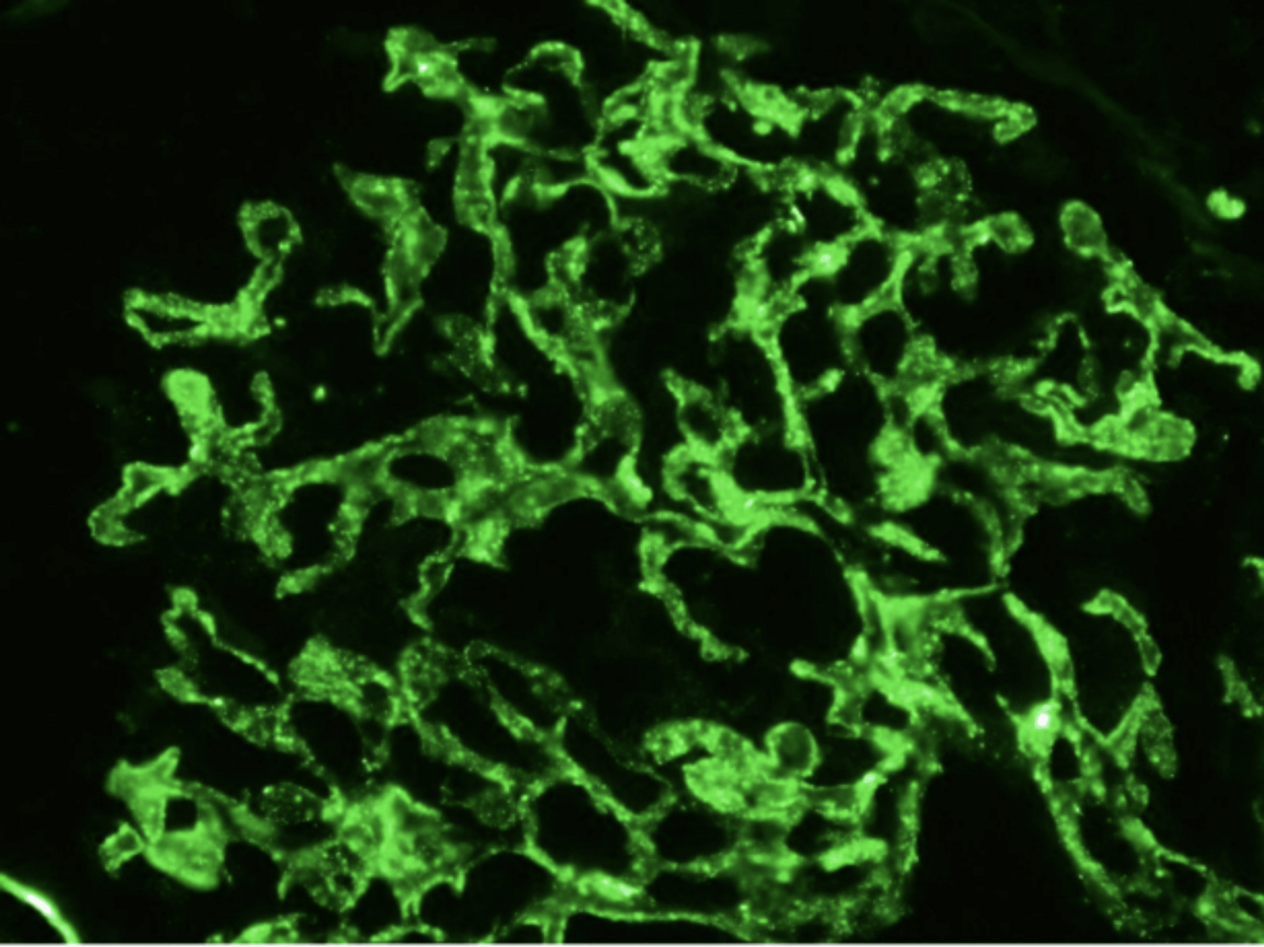
C3 in capillary loops as shown by immunofluorescence. The immunofluorescence image stains equally for IgG, IgM, kappa and lambda. However, C3 stains stronger and was dominate over the other markers.

 Based on the lab results, as seen above, the initial diuretic dose was increased, and a renal biopsy was ordered to assess the cause of her high proteinuria. The renal biopsy procedure went well with no complications. A detailed pathology report demonstrated membranous glomerulonephritis with PLA2R positivity and acute interstitial nephritis, as seen in the following images and pathology findings. For Figure 1, shown below, the renal parenchyma available for immunofluorescence studies is represented by approximately 80% cortex and 20% medulla. Fifteen glomeruli are present, nine of which are globally sclerotic. The sections are stained for IgG, IgM, IgA, C3, C1q, albumin, fibrinogen, and kappa and lambda light chains. There is global capillary wall finely granular staining for IgG (1+), IgM (1+), C3 (2+), kappa (1+), and lambda (1+). All other stains are negative within the glomeruli. There is no significant extraglomerular staining. Kappa and lambda stain equally throughout the tubulointerstitium.

The retrieved formalin-fixed paraffin-embedded tissue is stained for IgG, kappa, and lambda. There is capillary wall granular staining for IgG (1), kappa (1+), and lambda (1+). There is no significant extraglomerular staining. Kappa and lambda stain equally throughout the tubulointerstitium. A THSD7A immunofluorescence stain was performed, which was positive for the glomerular deposits. The performance of all immunofluorescence stains is verified for acceptability before results are reported. Internal antigens within the kidney parenchyma serve as positive and negative controls.

**Figure 2:**
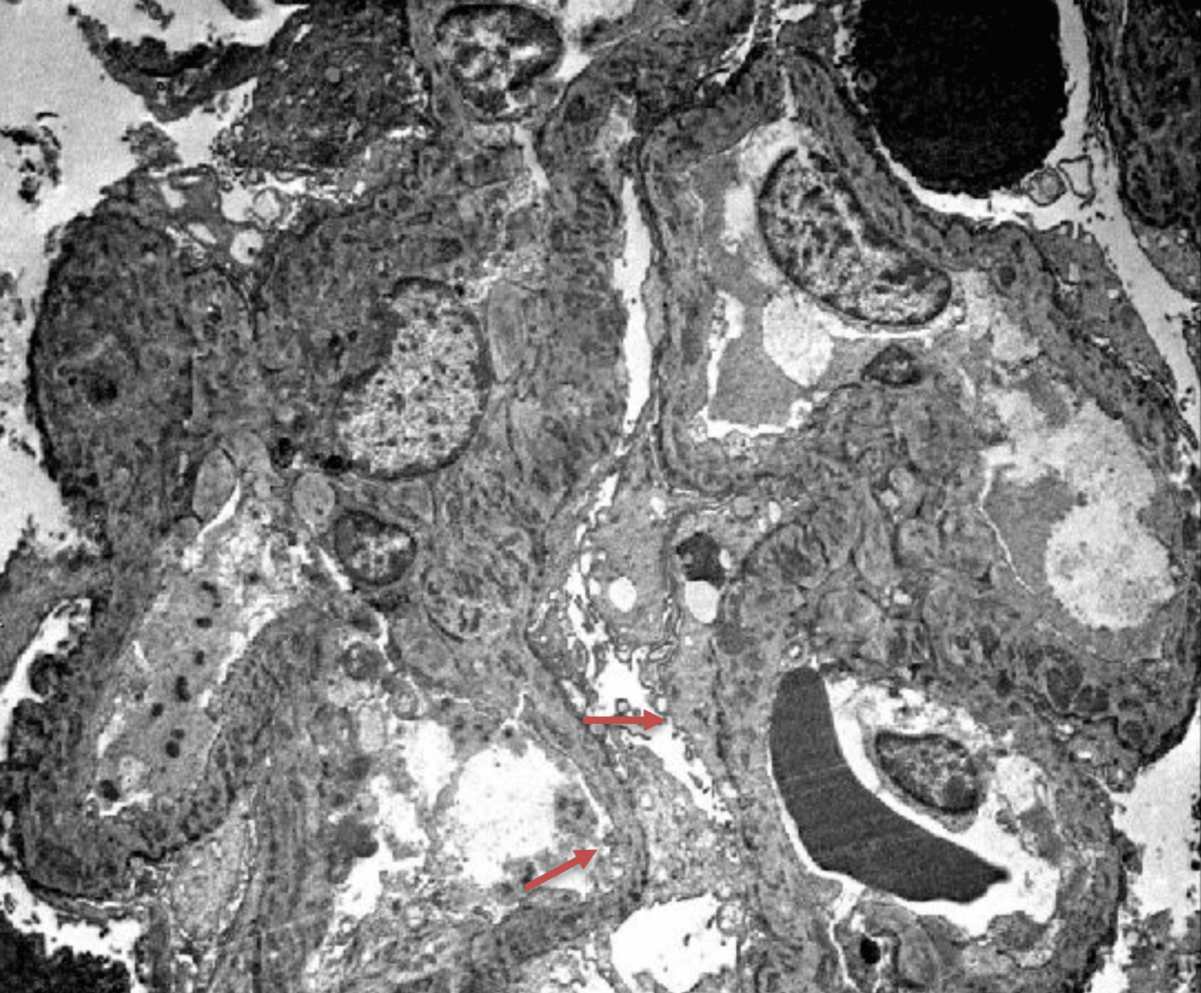
Intramembranous deposits as shown in electron microscopy. The electron microscope shows a build up of many small intramembranous immune deposits. Most of the epithelial foot processes have been damaged, as seen with the red arrows, and has contributed to the proteinuria as noted in the complete metabolic panel.

For Figure 2, shown below, two blocks are prepared. Ultrastructural examination of a glomerulus shows thickened glomerular basement membranes by frequent small intramembranous immune complex-type electron-dense deposits, some of which are partially resorbed. No subendothelial deposits are present. The epithelial foot processes are severely effaced. The mesangium shows few immune complex-type electron-dense deposits. The tubular basement membranes are without deposits. Anti-PLA2R Antibodies as a Prognostic Factor in PLA2R-Related Membranous Nephropathy (Figure 2). The results from the electron microscope demonstrated a thickened glomerular basement membrane with many small immune complex types, with a severe loss of foot process and anti-PLA2R antibodies as the prognostic factor in PLA2R-related membranous nephropathy. This confirmed the patient’s condition as primary membranous nephropathy.

**Figure 3:**
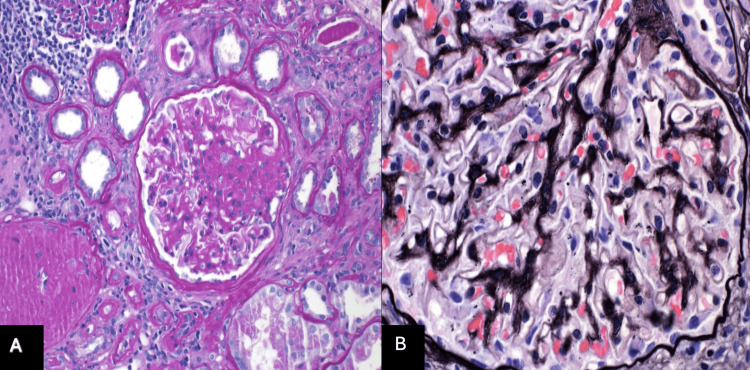
A: Segmental glomerulosclerosis as shown in light microscopy. B: Loops with minute holes as shown in light microscopy. Figure A shows areas of segmental sclerosis with interstitial fibrosis demonstrate lymphoplasmacytic inflammation. Figure B shows a globally sclerotic glomeruli with thickened capillary loops and minute holes using silver stain.

Light microscopy tissue sections were stained with Hematoxylin and Eosin, Periodic Acid Schiff, Jones Methenamine Silver, Silver Methenamine with Masson Trichrome, and Masson Trichrome. The renal parenchyma available for light microscopic examination was represented by approximately 50% cortex and 50% medulla. For Figure 3, shown below, ten glomeruli were present, six of which were globally sclerotic. The non-sclerotic glomeruli were enlarged and showed mildly thickened capillary loops with minute holes seen on silver stain. Three of the four non-globally sclerotic glomeruli show areas of segmental sclerosis. Per pathology, there was no endocapillary hypercellularity, fibrinoid necrosis, or cellular crescent formation. Severe interstitial fibrosis and tubular atrophy are present, involving approximately 70% of the cortical surface. Mild lymphoplasmacytic inflammation was seen within the areas of interstitial fibrosis. The non-atrophic tubules are lined by an attenuated, vacuolated, and reactive epithelium with loss of the proximal tubule brush borders. The arteries show moderate intimal fibrosis. The arterioles show moderate hyalinosis. PLA2R and EXT2 immunoperoxidase stains are performed on the frozen tissue and are negative within the glomerular deposits. Two toluidine blue-stained thick sections are prepared and show two open glomeruli."

Jones silver stain was used to evaluate the thickening, reduplication, spiking, or bubbling of the glomerular basement membrane. Controls were routinely run on all special stains and verified for acceptability. Before results were reported, the technical quality of routine slides was reviewed.

After the biopsy, the patient had a three-day stress dosing of solumedrol IV and was then transferred to a 60mg dose of prednisone. Creatinine peaked at 2.3 mg/dL during her hospital stay but then decreased to 1.9 mg/dL. She had persistent lactic acidosis, which was corrected with 1950mg twice daily of sodium bicarbonate and bistro 30mg twice daily. Potassium levels were monitored and were replaced appropriately during her stay. Lasix was initially held due to the risk of dehydration and rising creatinine levels but was eventually restarted at a low dose. The patient reported no urinary problems, and hemoglobin was stable during her stay. She was discharged shortly after. With this diagnosis, secondary causes were investigated, such as the patient’s HIV medicine. Discussion with the pharmacist concluded that the patient’s current HIV medications had no role in her kidney failure. The patient continued to follow-up at the nephrology clinic for regular follow-ups, and tacrolimus and cyclophosphamide were prescribed to halt renal failure. However, it was evident that the patient’s renal function was declining steadily. The patient eventually progressed to end-stage renal disease (ESRD) and was transferred to peritoneal dialysis. 

## Discussion

When it comes to the idiopathic causes of membranous nephropathy, it is hard to pinpoint what underlying condition leads to it. While it is known that patients are more likely to develop idiopathic membranous nephropathy due to autoantibodies against the PLA2R protein, we are unsure what purpose the PLA2R protein serves in the glomerulus itself [4]. While we initially thought that the patient's past medical history of HIV and antiretroviral medications could have led to her condition, discussions with the pharmacist suggested otherwise. Also, the positive anti-PLA2R antibodies found on the biopsy and in the pathology report identified this as a primary membranous nephropathy.

Patients with the anti-PLA2R autoantibody, as in our patient, is a marker to consider with regards to the pathophysiology behind idiopathic causes. It is thought that the amount of antibodies determines the outcome, with most idiopathic cases having anti-PLA2R [5]. The higher the autoantibody rate, the more likely a patient will progress through renal failure. Biopsy results can show the progressive buildup of the glomeruli either through immunofluorescence or immunohistochemistry [5]. This is noted in Figure 1, which shows the glomeruli covered in a green fluorescent color, which is indicative of a buildup, particularly that of C3. The immune deposits in the glomeruli resulted in impaired kidney filtration due to the gaps made, leading to increased proteinuria and other nephrotic-related symptoms, as in our patient [5]. This is noted in Figure 2, which shows the biopsy under an electron microscope of all the immune deposits built up.

In our case, our patient had nephrotic range proteinuria, as seen in Table 2, which prompted a kidney biopsy to establish the pathology since such large amounts of proteinuria with no history of pre-existing renal conditions made a biopsy the most appropriate next step. This was justified by the urine protein level of 500 mg/dL. It is also important to note that the GFR of 33.90 mL/min, as seen in Table 1, was evident of kidney failure, which played a role in performing the biopsy. The findings from the biopsy support the symptoms that the patient was dealing with and what we expected to find in a patient with primary membranous nephropathy. The light microscope images in Figure 3 also showed many holes and segmental glomerulosclerosis. That in of itself was evident of long-term progression [1]. The electron microscopy image in Figure 2 shows the loss of foot process, which is showed by the border being thinner than the the unaffected portion. These results played a major role in the patient’s proteinuria and that is what prompted us to do a biopsy. The immunofluorescence image in Figure 1 further proves this condition with IgG and C3 noted on the biopsy. The IgG and C3 are some of the biomarkers of that indicate membranous nephropathy. It was the positive stain for the anti-PLA2R antibodies that confirmed our diagnoses and guided our treatment options [1].

When it comes to the management and treatment of membranous nephropathy, it depends on how the disease will progress in the future, with patients with high-risk features being dealt with aggressively. Patients are considered at very high risk of progressive disease if they present with two or more of the following: a serum creatinine over 1.5mg/dL and higher, progressive decline in GFR by 25% over 2 years from the baseline or nephrotic syndrome defined by less than 2.5g/dL of albumin [6]. From these guidelines, our patient exhibits at least two of the criteria based on the patient's complete metabolic panel, as seen in Table 1, and that makes us consider using immunosuppressive therapy unless there is a contraindication, which there was not for our patient. The presence of anti-PLA2R antibodies further complicates the situation, encouraging us to be aggressive with the treatment options. The pathology report diagnoses this condition as membranous nephropathy by anti-PLA2R antibodies. With these risk factors and the autoantibodies, spontaneous remission is less likely in this case. 

When it comes to what immunosuppressive medications can be used, options such as rituximab or cyclosporine are considered [7], but tacrolimus and cyclophosphamide were selected. It is thought that patients with high-risk membranous nephropathy will benefit much more from either cyclophosphamide or chlorambucil, so cyclophosphamide was selected due to its milder side effects [8]. Tacrolimus was used in conjunction with cyclophosphamide for short-term benefits and worked alongside cyclophosphamide to preserve renal function [8]. While these drugs are well received in promoting remission, the anti-PLA2R antibodies make remission unlikely [8].

## Conclusions

Primary membranous nephropathy is a unique condition that can develop idiopathically and in which outcomes vary based on antibody development. In this condition, the PLA2R receptor is the focus as most cases due to primary causes have this receptor as the causative agent. What made this case interesting was the presence of anti-PLA2R antibodies that caused the patient’s symptoms, and it is what led to her eventual outcome. Overall, more testing and research needs to be done to better understand the PLA2 receptor and how we can prevent renal failure shortly after symptoms start.

While this case report gives a better understanding of membranous nephropathy associated with a positive PLA2R marker, there is more that we can learn about this condition. One such aspect is improved management of membranous nephropathy cases that involve high titer amounts of the autoantibody of PLA2R. It is a common theme that high antibodies mean the kidney will fail in the future. Initial steps should include studies on what exactly is PLA2R role in kidney pathology, better treatment options, possible risk factors such as HIV in our patient and how to better predict one's chance of getting this condition. By better understanding this receptor, we will have a better idea of what purpose it serves and develop medications that can potentially stop and reverse the damage that had been done.
